# The comprehensive detection of miRNA and circRNA in the regulation of intramuscular and subcutaneous adipose tissue of Laiwu pig

**DOI:** 10.1038/s41598-022-21045-2

**Published:** 2022-10-03

**Authors:** Hui Feng, Salsabeel Yousuf, Tianyi Liu, Xiuxiu Zhang, Wanlong Huang, Ai Li, Lingli Xie, Xiangyang Miao

**Affiliations:** State Key Laboratory of Animal Nutrition, Institute of Animal Sciences, Chinese Academy of Agricultural Sciences, Beijing, 100193 China

**Keywords:** Biochemistry, Computational biology and bioinformatics, Molecular biology, Zoology, Biomarkers, Diseases

## Abstract

circRNAs, as miRNA sponges, participate in many important biological processes. However, it remains unclear whether circRNAs can regulate lipid metabolism. This study aimed to explore the competing endogenouse RNA (ceRNA) regulatory network that affects the difference between intramuscular fat (IMF) and subcutaneous fat (SCF) deposition, and to screen key circRNAs and their regulatory genes. In this experiment, we identified 265 differentially expressed circRNAs, of which 187 up-regulated circRNA and 78 down-regulated circRNA in IMF. Subsequently, we annotated the function of DEcircRNA's host genes, and found that DEcircRNA's host genes were mainly involved in GO terms (including cellular response to fatty acids, lysophosphatidic acid acyltransferase activity, R-SMAD binding, etc.) and signaling pathways (fatty acid biosynthesis, Citrate cycle, TGF- β Signal pathway) related to adipogenesis, differentiation and lipid metabolism. By constructing a circRNA-miRNA network, we screened out DEcircRNA that can competitively bind to more miRNAs as key circRNAs (circRNA_06424 and circRNA_08840). Through the functional annotation of indirect target genes and protein network analysis, we found that circRNA_06424 affects the expression of *PPARD*, *MMP9*, *UBA7* and other indirect target genes by competitively binding to miRNAs such as ssc-miR-339-5p, ssc-miR-744 and ssc-miR-328, and participates in PPAR signaling pathway, Wnt signaling pathway, unsaturated fatty acid and other signaling pathways, resulting in the difference of fat deposition between IMF and SCF. This study provide a theoretical basis for further research investigating the differences of lipid metabolism in different adipose tissues, providing potential therapeutic targets for ectopic fat deposition and lipid metabolism diseases.

## Introduction

The amount and distribution of fat deposition in pigs are important factors affecting meat quality^1^. Intramuscular fat (IMF) refers to adipocytes embedded in porcine skeletal muscle tissue. IMF determines the flavor, tenderness and juiciness of the meat, and is an important indicator for the quality of meat^2^. Subcutaneous fat (SCF) mainly accumulates in the back and abdomen of pigs. SCF can affect the carcass traits of pigs and is negatively correlated with lean meat percentage^3^. In the breeding process, we expect to breed pigs with high lean meat percentage and high intramuscular fat percentage. It is necessary to reduce subcutaneous fat deposition and increase intramuscular fat deposition. However, there is a high degree of synergy between IM and SC fat deposition in pigs^4^. Therefore, identifying differences in metabolic regulation between adipocytes of the two origins can be helpful for the development of pig breeding that can independently manipulate IM and SC fat deposition.

Adipocyte metabolism is a complex process that is finely regulated by many factors, such as hormones, transcription factors signaling pathways, etc.^5^. Among them, both intramuscular and subcutaneous adipocytes belong to white adipocytes. They are highly similar in the process of differentiation and metabolism, but there are also many differences. Intramuscular adipocytes are mainly derived from bone marrow mesenchymal stem cells (MSCs)^6^ and mesenchymal progenitor cells with positive platelet-derived growth factor receptor α (*PDGFR α*)^7^. Stem cells (SCs), fibroadipocyte precursors (fFAPs), fibroblasts, myoendothelial cells, pericytes, hemangioblasts and *PW1* overexpressing cells have been found to serve as sources of intramuscular adipocytes^8,9^. 10–25% of adipocytes in human subcutaneous fat are derived from bone marrow progenitor cells^10^. During adipogenic differentiation, early recruitment of adipocytes is dependent on the regulation of typical Wnt and BMP4 signaling pathways. Wnt signaling pathway enhances the proliferation and differentiation of preadipocytes^11^, while*BMP4* induces the differentiation of preadipocytes into adipocytes^12^. Scholars found that the expression levels of key adipogenic genes, such as peroxisome proliferator-activated receptor gamma (*PPAR γ*) and fatty acid synthase (*FAS*) in intramuscular adipocytes were lower than that in subcutaneous adipocytes. This results in a weaker lipogenic capacity of intramuscular adipocytes than subcutaneous adipocytes^4,13,14^. Some studies have shown that some bioactive substances, such as conjugated linoleic acid, can reduce or maintain the deposition of subcutaneous fat, and increase the content of intramuscular fat^15^. Therefore, the biological function and molecular regulation mechanism of intramuscular fat deposition are different from that of subcutaneous fat. Some studies have shown that circRNA plays a potential regulatory role in the process of adipose metabolism^16^.

Circular RNA (circRNA), an RNA without 5 'and 3' polarity^17^, discovered as a class of molecules with functional relevance for the regulation of gene expression^18^. With the continuous development of high-throughput sequencing technology and bioinformatics, researchers have found that circRNAs are widely present in multicellular eukaryotes^19^, such as human (*Homo sapiens*)^20^, mouse (*Mus musculus*)^21^, drosophila (*Drosophila melanogaster*)^22^, Caenorhabditis elegans (*Caenorhabditis elegans*)^23^, zebrafish (*Danio rerio*)^24^, rice (*Oryza sativa*)^25^, etc. The circRNA sequences of human, mouse, nematode, speartail and coelacanth were included in the circBase database^26^. circRNA is widely present in mammals with the characteristics of long half-life, conserved sequence structure and tissue-specific expression. At present, the research on the biological function of circRNA have shown that circRNA can regulate the expression of miRNA and reduce the inhibitory effect of miRNA on target mRNA, mainly by acting as the molecular sponge of miRNA^27^. In addition, studies have shown that some circRNA can regulate regulate gene expression by interacting with RNA binding proteins (RBPs)^28^. A large number of studies have shown that circRNAs are related to many diseases, such as Alzheimer's disease^29^, cancer^30^, Cardiovascular and cerebrovascular^31^ and neurological disorders^32^; and the process of cell growth and development, for example, cell proliferation^33^, oogenesis^34^ and embryonic development^35^.

In this study, we selected Laiwu pigs with high-fat deposition capacity, and obtained their intramuscular fat and subcutaneous adipose tissue. By next-generation sequencing technology, circRNAs and miRNAs were identified in intramuscular and subcutaneous adipose tissue. Gene ontology (GO) and Kyoto Encyclopedia of Genes and Genomes (KEGG) were also used for functional enrichment analyses, circRNAs and miRNAs involved in the regulation of genes related to fat development in Laiwu pigs were identified. We further studied adipogenic gene expression patterns and their enriched pathways in Laiwu Pig to better understand their regulatory roles in adipogenic differentiation and fat metabolism.

## Materials and methods

### Ethics statement

All procedures involving animals were approved by the Animal Care and Use Committee at Institute of Animal Sciences (Chinese Academy of Agricultural Sciences), where the experiments were performed. All experiments were performed in accordance with guidelines and regulations formulated by the Ministry of Agriculture of the People’s Republic of China.

### Experimental animals and sample preparation

Three 180-day-old Laiwu sows of similar body weight were killed by exsanguination. The subcutaneous fat and intramuscular adipose tissue at the longissimus dorsi muscle were quickly stripped and put into the pre-labeled cryopreservation tubes (L_PX_1, L_PX_2, L_PX_3 and L_JN_1, L_JN_2, L_JN_3. L_PX represents the subcutaneous adipose tissue of Laiwu pigs, and L_JN represents the intramuscular adipose tissue of Laiwu pigs). Then, the cryopreservation tubes were placed in liquid nitrogen. Then, they were transported back to the laboratory with dry ice.

### RNA isolation and circRNA sequencing

We placed the adipose tissues in a mortar pre-cooled with liquid nitrogen and quickly ground it to powder. In order to obtain total RNA, we sequentially added 1 mlTRlzol((Invitrogen Life Technologies, Carlsbad, USA), 0.2 ml chloroform, 0.2 ml isopropanol, 75% ethanol and RNase-free water. The integrity of sample RNA was preliminarily determined by 1% agarose gel electrophoresis. To ensure RNA concentration, we used Nanodrop to detect od260 / 280 and od260 / 230 values. We accurately evaluated the integrity, purity and degradation degree of RNA through Aglient 2100 Bioanalyzer. Six cDNA libraries (L_PX_1, L_PX_2, L_PX_3 and L_JN_1, L_JN_2, L_JN_3) were constructed through TruSeq Stranded Total RNA LT Kit with Ribo-Zero TM Gold (RS-122-2301). The libraries building process includes mRNA enrichment, fragmentation, reverse transcription, double-stranded cDNA synthesis, linker, DNA purification and PCR amplification. To test the length and quality of the library, we added samples to the Agilent 2100 biological analyzer. After the quality inspection is qualified, samples were sequenced by Illumina HiSeqTM2500 platform.

### circRNA identification and differential analysis

We first adopted paired-end sequencing. After obtaining sequencing data, we used NGSQCToolkit (v2.3.3) for quality control and adapter trimming (Keep default parameters). Then, low-quality bases and N-bases were trimmed from the reads. Finally, high-quality clean reads were obtained for subsequent analysis. CIRI^36^ was used to identify circRNAs. DEseq2^37^ was used for differential expression analysis of circRNAs. |log2FoldChange|≥ 1 and P_value ≤ 0.05 were used to indicate significant difference in circRNA expression between treatment groups and the control group. Using the intersectbed software^38^, we obtain the protein-coding transcript with the largest overlapping region of circRNA in the genome, that is, the host gene of circRNA, based on the number of cyclization sites in the transcript region and the number of exons between the shear sites.

### Bioinformatics analysis and target gene prediction

We performed miRNA target prediction for circRNAs using MiRanda software^40^. To explore the potential functions of circRNAs in adipose tissue, we selected circRNAs that can competitively bind more miRNAs as key circRNAs, and analyzed the sequence of key circRNAs. The open reading frames (ORFs) of circRNA were predicted by ORFinder (http://www.bioinformatics.org/sms2/orf_find.html). The prediction results were input into the Conserved Domains database (https://www.ncbi.nlm.nih.gov/Structure/cdd/wrpsb.cgi) to predict the protein-coding ability of ORFs of circRNA.

### GO and KEGG enrichment and protein–protein interaction network analysis

We performed GO (gene ontology, http://www.geneontology.org) and KEGG (Kyoto encyclopedia of genes and genomes, http://www.genome.jp/kegg) enrichment analysis on the host and indirect target genes of circRNA using clusterProfiler package^41^ in R software, and P_value ≤ 0.05 was significant enrichment. The enrichment results were shown in bar graphs and bubble graphs.

We selected circRNAs that can bind to more miRNAs as key circRNAs. Taking the differentially expressed genes targeted and regulated by the key circRNA, we applied the STRING database to analyze the protein interaction network. Then, we input the prediction results with a comprehensive score greater than 0.4 (medium) into the cytoscape software for visual display.

### Validation of real-time fluorescence quantitative PCR

6 circRNAs (circRNA_00907, circRNA_01106, circRNA_06424, circRNA_08840, circRNA_15332 and circRNA_22410), 3 miRNAs(miR-328, miR-361-3p and miR-331-3p) and 2 genes(*PPARD* and *MMP9*) were randomly selected for qRT-PCR validation experiment to ensure the accuracy of sequencing data. qRT-PCR was performed in triplicate, and total RNA was extracted from tissue samples using the Total RNA Extraction Kit (DNase I) kit (GenePool, Cat# GPQ1801). Then, reverse transcription was performed with circRNA cDNA Synthesis Kit (GenePool, Cat# GPQ1805), miRNA cDNA Synthesis Kit (GenePool, Cat# GPQ1804) and mRNA cDNA Synthesis Kit (GenePool, Cat# GPQ1803). We performed quantitative analysis of data through Bioer linegene 9600plus fluorescence quantitative PCR instrument and 2^−△△Ct^ method^42^, detailed in Table 1. At the same time, the melting curve analysis was performed at 60–95 °C, and the standard curve of the target gene was drawn.

**Table 1:** Gene and the corresponding primer sequences.

Gene	Forward primer	Reverse primer
circRNA_00907|1:93126380_93142829_+	AGAGTGAGAGGTCCTGAATATGATG	CACCACGACAGCCTTGATGA
circRNA_01106|1:110525008_110548336_+	ACCTGGAGATGCTGTTCATAAGAT	CGGCTAATGTTGCAAGGGAAAT
circRNA_06424|12:62458832_62523689_−	GAGCAGAAGCACCGAAGTATTG	TGAACTTGACATTCTCAGGACAGA
circRNA_08840|13:217685619_217779051_−	TGTGTTCCTAGATGGAGCCTCT	CGTGGTTAGTTTCTGCGTTGG
circRNA_15332|2:59187624_59255661_+	GATGAGACCAACGACGAGGAG	TCAGAGCTGCTGTAGACCTTG
circRNA_22410|6:92745097_92803482_+	GGCATCTTGCTGTTCGTGATTA	ATGGTCTCCTTCCGCTTCTTG
miR-328	CCCTCTCTGCCCTTCCGT	
miR-361-3p	CCAGGTGTGATTCTGATTTGC	
miR-331-3p	CCCCTGGGCCTATCCTAGAA	
PPARD	CGAGTTCGCCAAGAGCATCC	GCACGCCGTACTTGAGAAGG
MMP9	TGTTAAGGAGCACGGAGATGG	TGGCGGTCGGTGTCATAGT

### miRNA and mRNA sequencing

We obtained the seqencing results of circRNA, miRNA and mRNA in the same experiment. The results of miRNAs and mRNAs have been published in "Frontiers in Veterinary Science" (https://www.frontiersin.org/articles/10.3389/fvets.2022.976603/full).

### Statistical analysis

All data are presented as mean ± standard. A student’s t-test was performed for comparisons by excel, p < 0.05 was statistically significant.

### Ethics approval

The experimental procedure was approved and supervised by the Animal Care Commission of the Institute of Animal Sciences, Chinese Academy of Agricultural Sciences. All experiments were performed in accordance with guidelines and regulations formulated by the Ministry of Agriculture of the People’s Republic of China.

## Results

### Reads mapping transcriptome and quality control

**Figure 1:**
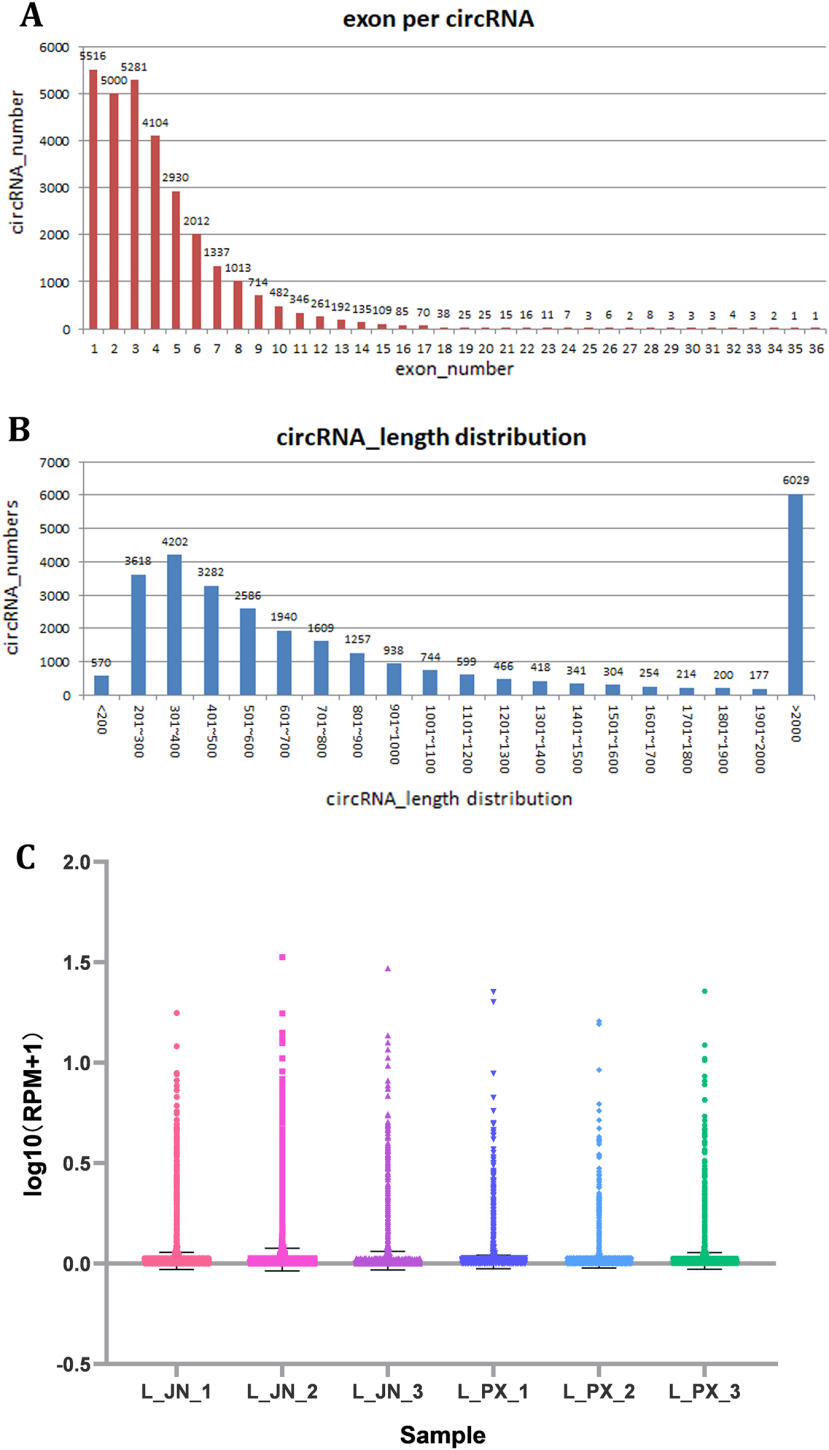
Overview of circRNAs in the L_JN and L_PX. (**A**) circRNA length distribution. (**B**) Exon content. (**C**) Expression level.

The original data in L_PX and L_JN were subjected to quality control, and the clean reads results were shown in Table 2. Q30 ≥ 90% and GC ≥ 61% in 6 samples indicated reliable sequencing results. We compared clean reads with reference genes, and identified 29,763 circRNAs. The length distribution of circRNAs sequences and the number of exons were shown in Fig. 1A,B, receptively. The length of these circRNAs sequences was mainly between 201 and 600 bp, and the content of exons was mainly from1 to 5. Based on rpm values of circRNAs expression, we constructed an RPM box Whistler plot, as shown in Fig. 1C. The expression of circRNAs in intramuscular adipose tissue was higher than that in subcutaneous tissue.

**Table 2:** Summary of reads of Laiwu Pig transcriptome.

Sample	Raw reads	Clean reads	Valid bases (%)	Q30 (%)	GC (%)
Sample_L_JN_1	80013516	77555412	96.76	92.25	63
Sample_L_JN_2	79818561	77374868	96.78	92.37	61
Sample_L_JN_3	79623606	77194324	96.80	92.49	59
Sample_L_PX_1	79432576	75995332	95.46	90.38	66.50
Sample_L_PX_2	79473958	75914582	95.28	90.44	66.50
Sample_L_PX_3	79526364	76470692	95.98	91.52	61.50

L_JN_1-3 refers to intramuscular adipose tissues; L_PX_1- 3 refers to subcutaneous adipose tissues.

### Identification of differentially expressed circRNA

**Figure 2:**
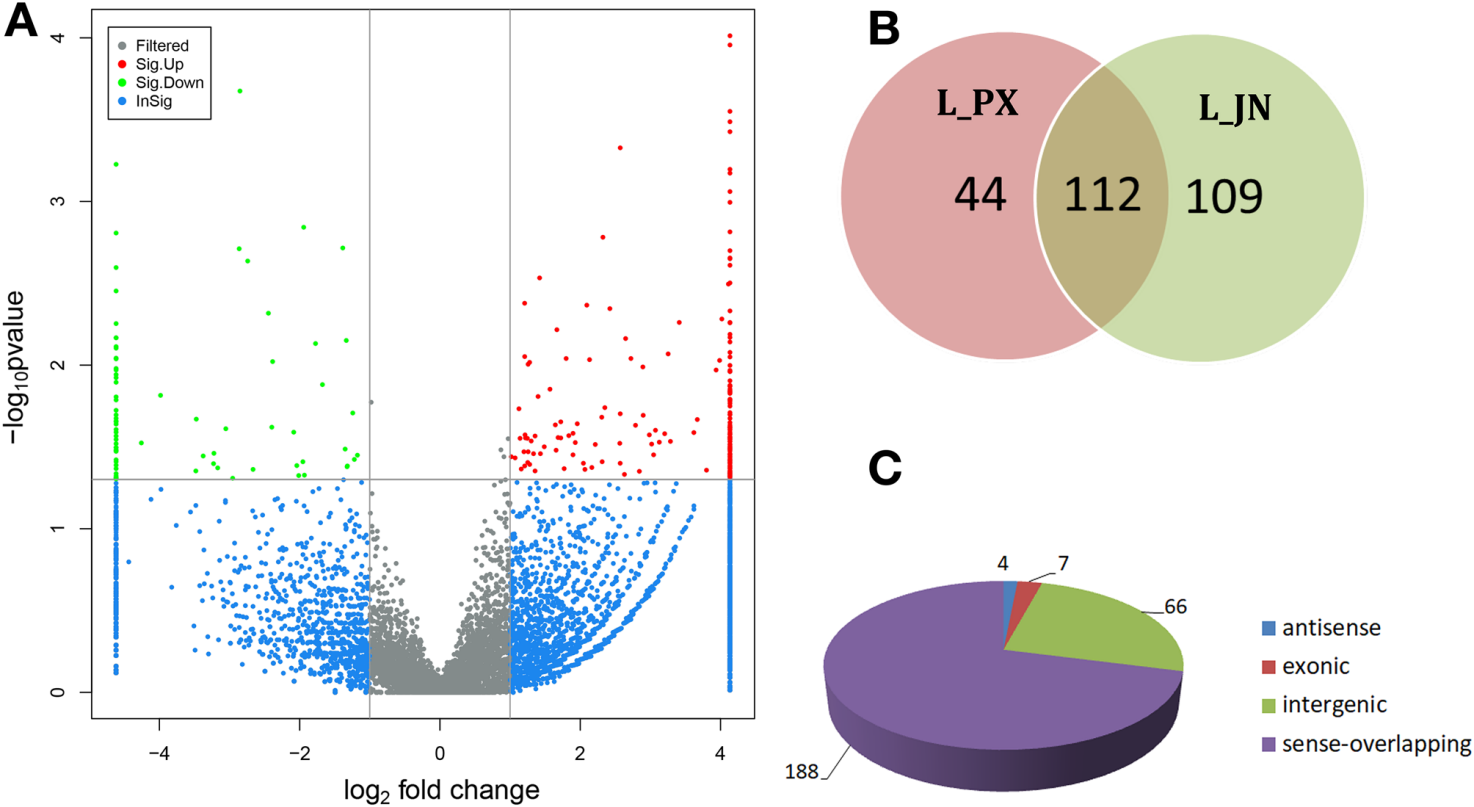
Overview of DEcircRNAs in the L_JN and L_PX. (**A**) Volcano plots showing the distribution of differentially expressed circRNAs between two adipose tissues. (**B**) Specific expression circRNA. (**C**) The proportion of various circRNAs in total DEcircRNAs.

There are significant differences between subcutaneous fat and intramuscular fat deposition of pigs, and the expression of circRNAs is tissue-specific. Through the differential expression analysis of circRNA, we screened 265 differentially expressed circRNAs, including 187 up-regulated and 78 down regulated in IMF. The differential expressions of 104 DEcircRNAs were more than 4 times, as shown in Fig. 2A. The specific expression of DEcircRNA was analyzed. 44 DEcircRNAs were specifically expressed in L_PX, 109 DEcircRNAs were specifically expressed in L_JN, and 112 DEcircRNAs were expressed in both tissues, as shown in the Venn diagram of Fig. 2B. 4 antisense, 7 exonic, 66 intergenic and 188 sense-overlapping circRNAs identified among 265 DEcircRNAs, as shown in Fig. 2C.

### Functional annotation of circRNA host genes

**Figure 3:**
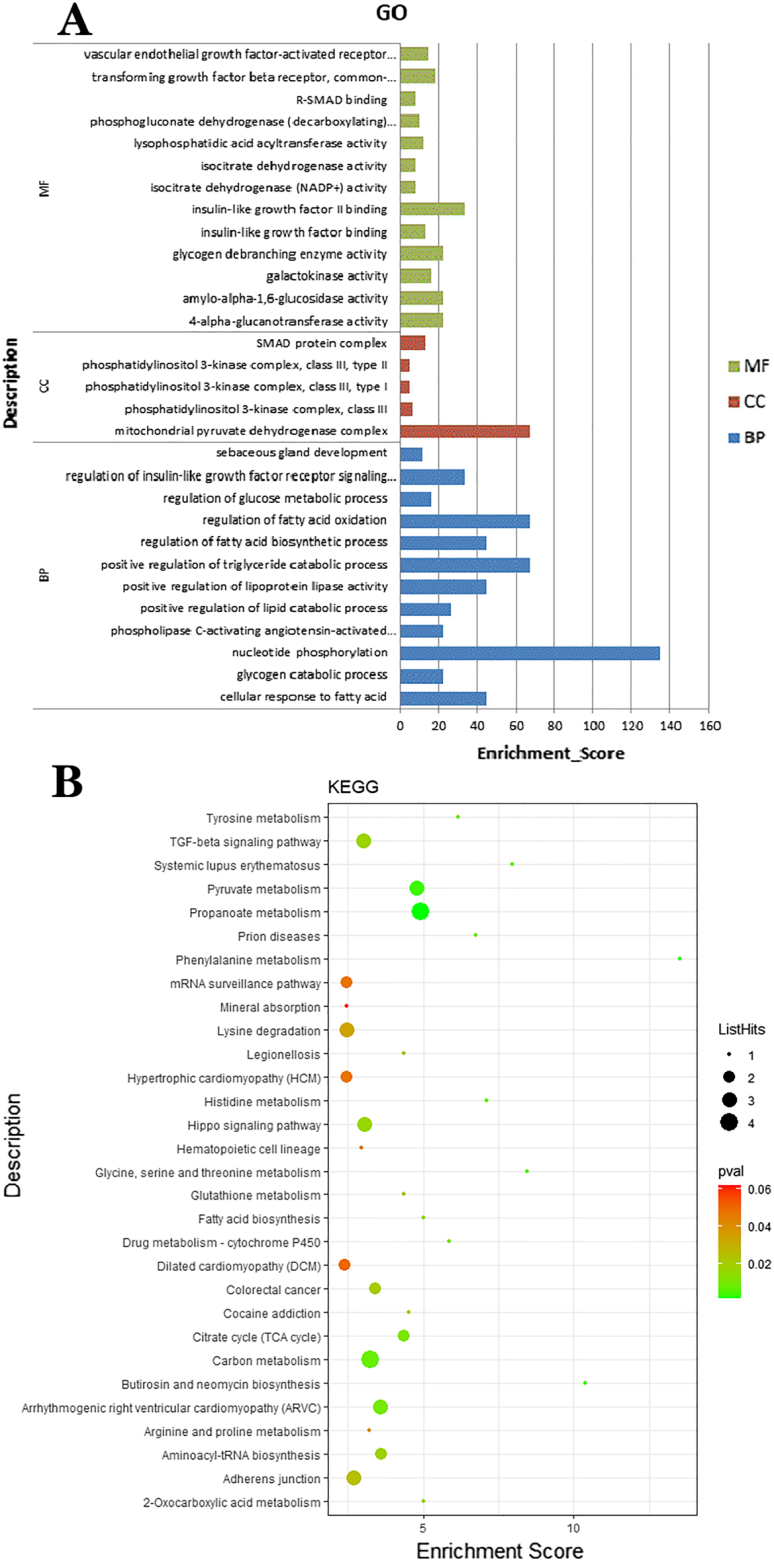
Function annotation of host genes. (**A**) GO enrichment analysis. (**B**) KEGG pathway analysis. The x-axis represents the enrichment_score, the y-axis represents the name of the GO terms or pathways.

A large number of studies have shown that circRNA can affect the transcription of host genes. Therefore, we performed GO and KEGG (Fig. 3) enrichment annotations of the host genes of DEcircRNAs. The host genes of DEcircRNAs were significantly involved in 638 GO terms, of which a large number of GO terms were related to fat deposition and metabolism (Fig. 3A). In biological process, host genes were mainly involved in cellular response to fatty acid, glycogen catabolic process, nucleotide phosphorylation, phospholipase C-activating angiotensin-activated signaling pathway, positive regulation of lipid catabolic process. From the perspective of cell composition, host genes are mainly involved in mitochondrial pyruvate dehydrogenase complex, phosphatidylinositol 3-kinase complex, class III, phosphatidylinositol 3-kinase complex, class III, type I, phosphatidylinositol 3-kinase complex, class III, type II, SMAD protein complex. From the perspective of molecular functions, host genes are mainly involved in 4-alpha-glucanotransferase activity, amylo-alpha -1,6-glucosidase activity, galactokinase activity, glycogen debranching enzyme activity, etc. The differentially expressed circRNA was significantly involved in 28 signal pathways with P-value ≤ 0.05 (Fig. 3B). Among them, the Citrate cycle (TCA cycle), Propanoate metabolism, Phenylalanine metabolism, Pyruvate metabolism, Fatty acid biosynthesis, TGF- Beta signaling pathways and other signaling pathways are already proven as signaling pathways related to fat deposition and metabolism.

### Bioinformatics analysis and target gene prediction

**Figure 4:**
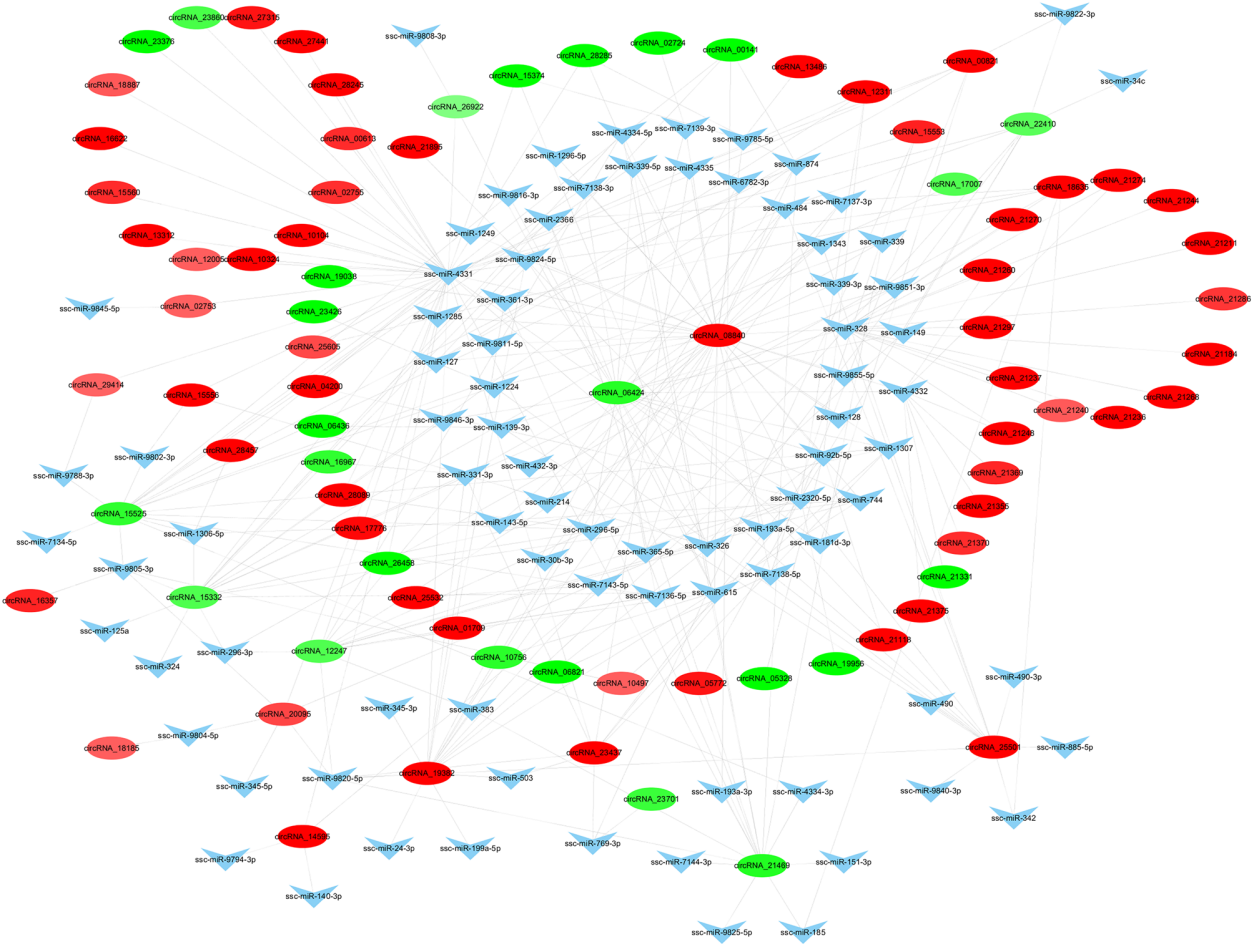
circRNA-miRNA interaction network. The circle represents the circRNA; triangle represents the miRNA; red represents upregulation; green represents downregulation; blue represents no difference.

As a non-coding RNA, current research shows that circRNA plays a regulatory role mainly through competitive binding of miRNAs, thereby affecting the biological functions of miRNAs. Therefore, the miRNA regulated by DEcircRNA is important in this experiment. The target miRNAs of DEcircRNAs were predicted using miRanda software. Taking the circRNA-miRNA relationship pair of top300, we used the cytoscape software to draw the circRNA-miRNA regulatory network diagram. In Fig. 4, circRNA_08840 can bind 43 miRNAs, and circRNA_06424 can bind 25 miRNAs, which are the two circRNAs that can bind the most miRNAs. So the down-regulated circRNA_06424 and the up-regulated circRNA_08840 in the regulatory network can serve as molecular sponges for more miRNAs with a key role in adipogenic differentiation and lipid metabolism. In addition, DEcircRNAs, such as circRNA_19382, circRNA_15332, circRNA_15525, circRNA_21469 can also regulate many miRNAs.

### Key circRNAs sequence analysis

Several human circRNAs had translation capabilities and played a key role in tumors and cancers^43^. By constructing the circRNA-miRNA regulatory network, we screened the key circRNA_06424 and circRNA_08840. We found that circRNA_06424 and circRNA_08840 are both sense-overlapping circRNAs, which are transcribed from gene regions with potential coding capabilities. circRNA_06424 has a sequence length of 64858 bp and is located on pig chromosome 12, derived from *ENSSSCG000000180391*. circRNA_08840 has a sequence length of 93433 bp, is located on chromosome 13 of pigs, and derived from *ADARB*. The results of ORFinder Prediction shows that there are 254 and 342 open reading frames (ORFs) in circRNA_06424 and circRNA_08840, respectively. We predicted the potential coding ability of Conserved Domains for these ORFs. The results are shown in Tables 3 and 4.

**Table 3:** circRNA_06424 sequence ORFs coding ability prediction information.

Query	PSSM-ID	From	To	E-Value	Bitscore	Accession	Short name
ORF 198	240654	3	36	0.005757	31.5226	cd12177	2-Hacid_dh_12
ORF 198	419666	3	36	0.005757	31.5226	cl21454	NADB_Rossmann superfamily
ORF 222	223021	21	112	0.008785	36.0697	PHA03247	PHA03247
ORF 222	223021	21	112	0.008785	36.0697	cl33720	PHA03247 superfamily

**Table 4:** circRNA_08840 sequence ORFs coding ability prediction information.

Query	PSSM-ID	From	To	E-Value	Bitscore	Accession	Short name
ORF 62	237973	2	33	0.002159	32.3316	cl36492	PRK15488 superfamily
ORF 80	223021	3	291	0.009692	37.9957	cl33720	PHA03247 superfamily
ORF 128	237865	97	206	0.001266	40.0828	cl36447	PRK14951 superfamily
ORF 216	411326	41	67	0.00088	38.5856	cl41408	NlpC_p60_RipA superfamily
ORF 232	419665	1	30	0.001868	32.6703	cl21453	PKc_like superfamily
ORF 245	413469	17	58	0.002999	34.0771	cl02775	Oxidoreductase_nitrogenase superfamily

### Competing endogenouse RNA (ceRNA) network

**Figure 5:**
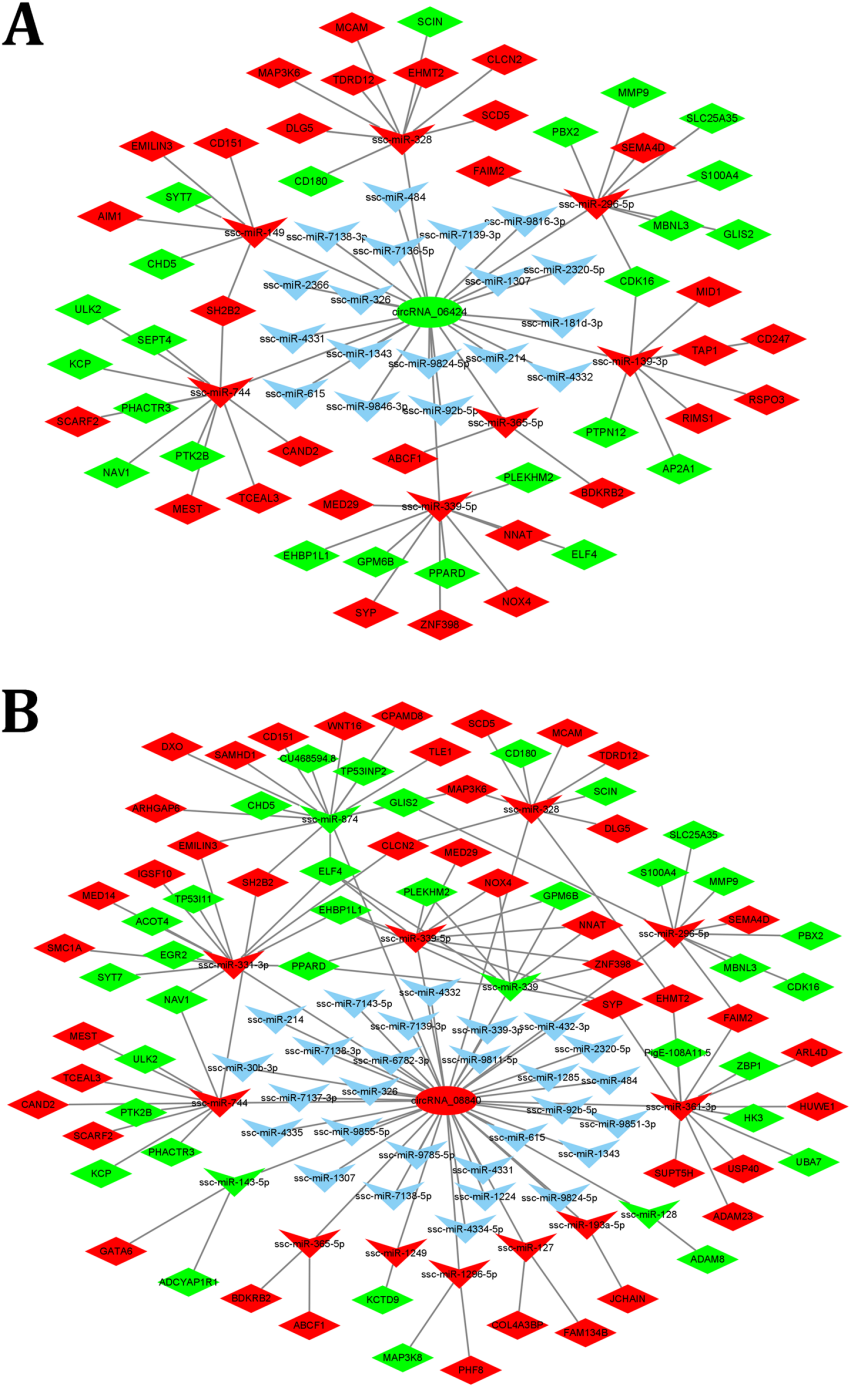
ceRNA networks of (**A**) circRNA_06424 and (**B**) circRNA_08840. Circle represents circRNA; triangle represents the miRNA; diamond represents gene; red represents up-regulation; green represents down-regulation; blue represents no difference.

circRNA binds miRNA competitively with mRNA, thereby reducing the inhibitory effect of miRNA on mRNA, and increasing the expression of indirect target genes. The ceRNA regulatory network is usually constructed by DEcircRNA-DEmiRNA-DEgene to explore the function of circRNA. In this study, we selected circRNA_06424 and circRNA_08840 target miRNAs with higher expression and its target DEgenes, to construct a ceRNA network (Fig. 5). In the ceRNA network centered on circRNA_06424, there are 7 down-regulated DEmiRNAs and 52 DEgenes with higher expression levels. In the ceRNA network centered on circRNA_08840, there are 8 DEmiRNA and 44 DEgene. circRNA_06424 and circRNA_08840 have three common target miRNAs (ssc-miR-339-5p, ssc-miR-744, ssc-miR-328).

### Function annotation of intircet target genes

**Figure 6:**
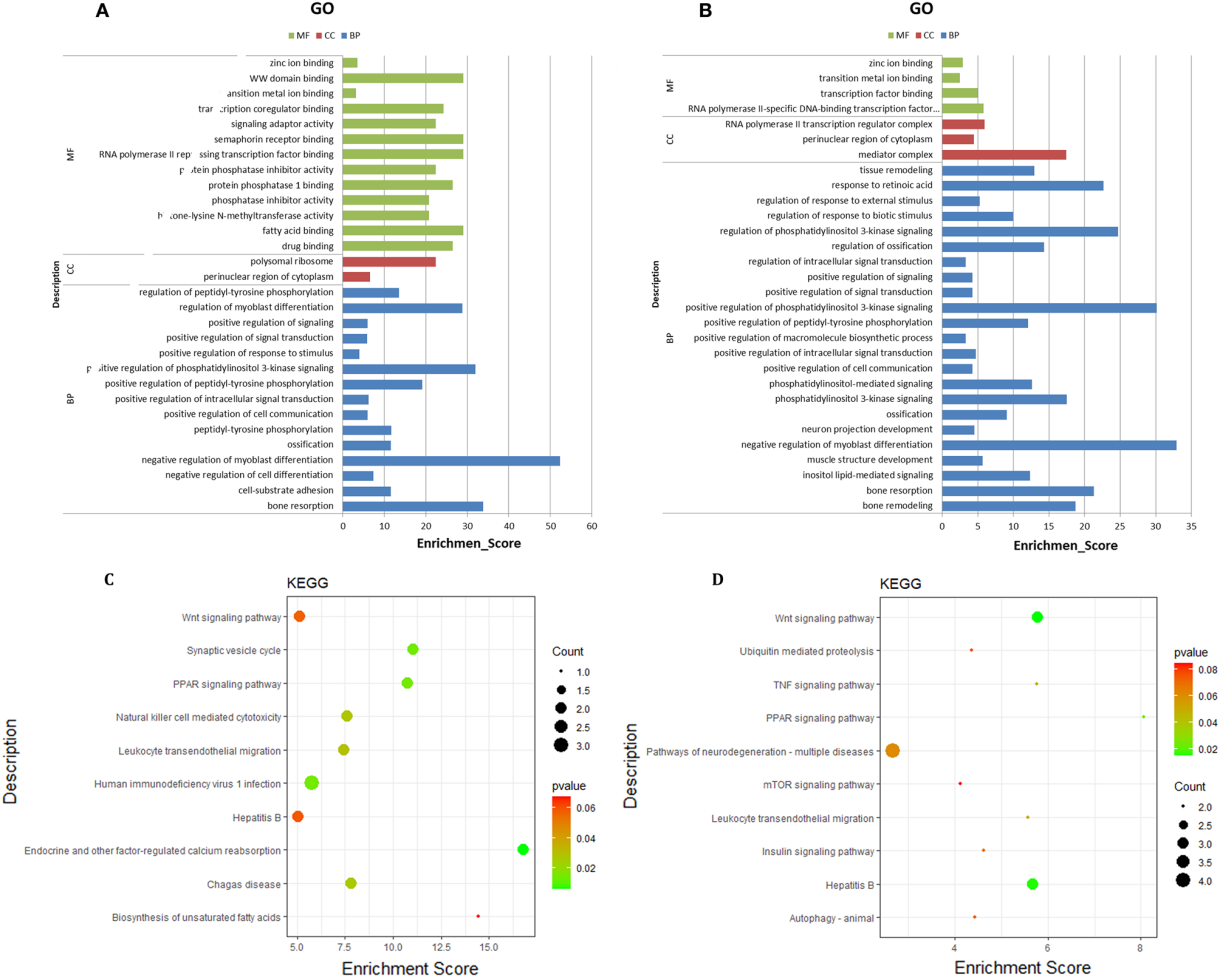
GO analysis of target genes of (**A**) circRNA_06424 and (**B**) circRNA_08840. KEGG parhway analysis of target genes of (**C**) circRNA_06424 and (**D**) circRNA_08840.

We found the genes targeted and regulated by the key circRNA through the construction of the ceRNA network. To further explore the function of circRNA, we performed GO and KEGG enrichment analysis on the indirect target genes of the key circRNAs. The indirect target genes of CircRNA_06424 were significantly involved in 160 GO terms, and the top30 GO terms were selected, as shown in Fig. 6A. Among them, GO terms related to fat deposition and metabolism are fatty acid binding, phosphatase inhibitor activity, positive regulation of phosphatidylinositol 3-kinase signaling. There are GO terms related to cell differentiation, such as negative regulation of myoblast differentiation. In Fig. 6C, KEGG enrichment analysis revealed that the indirect target genes of circRNA_06424 are involved in the signaling pathways related to lipogenesis and metabolism in Biosynthesis of unsaturated fatty acids, PPAR signaling pathway, and Wnt signaling pathway. In addition, a large number of studies have shown that lipid production and metabolism will also change in patients with immune reactions. Therefore, signal pathways such as Chagas disease, Human immunodeficiency virus 1 infection, and Hepatitis B also need attention in the difference between subcutaneous and intramuscular fat deposition. The indirect target genes of circRNA_08840 were significantly involved in 134 GO entries. The top 30 GO terms were selected, as shown in Fig. 6B. Many of these GO terms were related to fat deposition and metabolism, such as positive regulation of phosphatidylinositol 3-kinase signaling, inositol lipid-mediated signaling, negative regulation of myoblast differentiation. The KEGG results (Fig. 6D) showed that these indirect target genes are involved in Wnt signaling pathway, Insulin signaling pathway, PPAR signaling pathway, and some disease-related signaling pathways, such as Hepatitis B, TNF signaling pathway, mTOR signaling pathway.

### Protein–protein interaction (PPI) network

**Figure 7:**
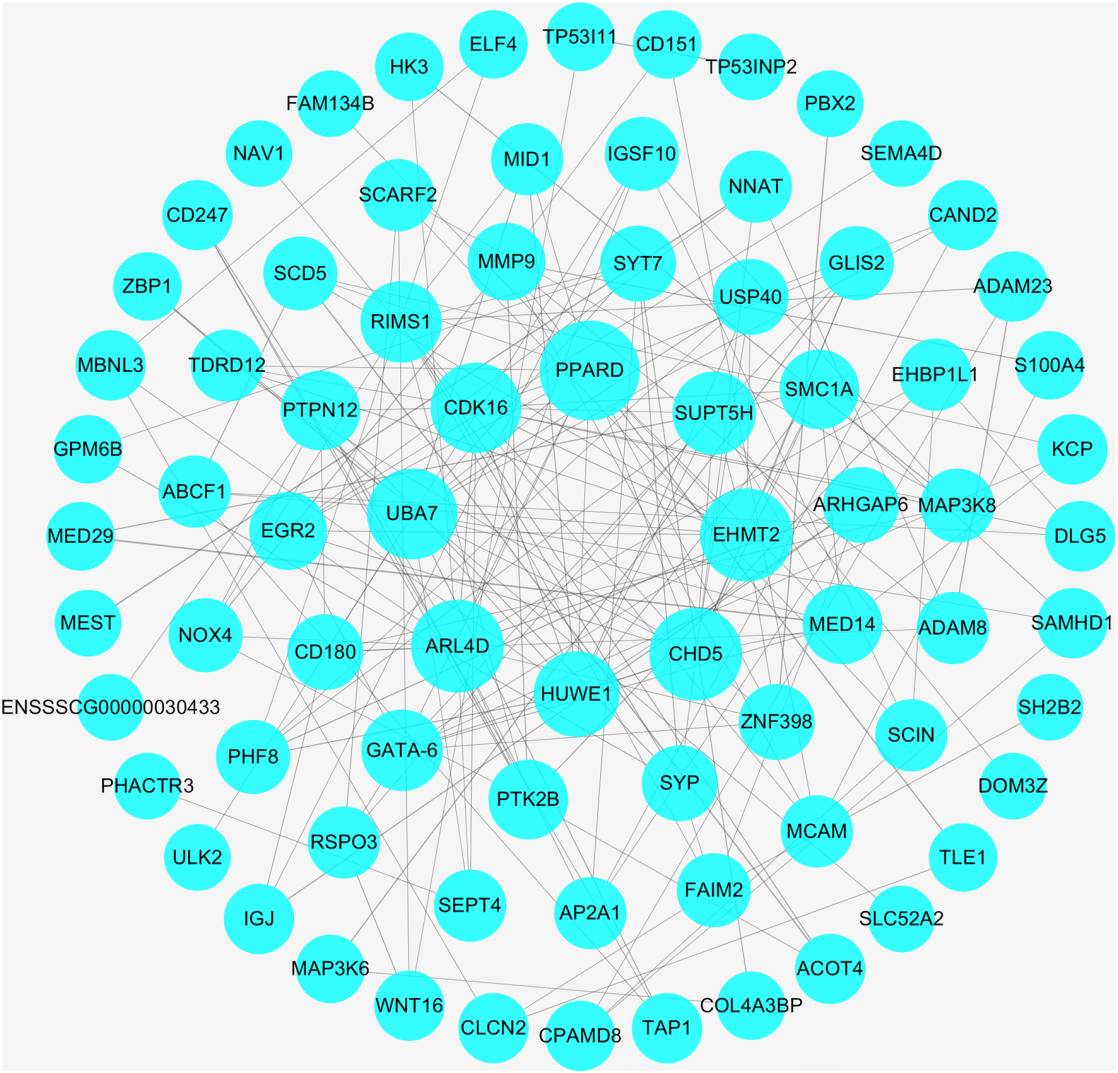
Protein–protein interaction network between top 300 downregulated and up-regulated DEGs in L_JN and L_PX by threshold p-value < 0.05, |log2fold change|≥ 1. The node size represents degree.

Studying the interaction network between proteins can find out the core regulatory genes. In this experiment, we studied the protein interaction relationship between the key circRNA indirect target genes identified in L_JN and L_PX, and used the degree value of each protein as a screening index to draw a PPI network diagram, as shown in Fig. 7. *PPARD*, *CHD5*, *CDK16*, *UBA7*, *EHMT2*, *ARL4D*, *SUPT5H* and *HUWE1* are at key nodes of the PPI network, and can interact strongly with more proteins. This indicates that these proteins can play an important role in the significant difference between IMF and SCF deposition levels.

### qRT-PCR verification

**Figure 8:**
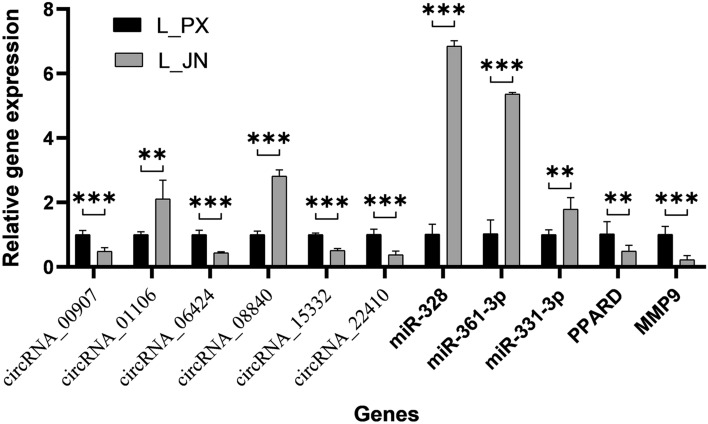
qRT-PCR verification results. The differential expression of genes between intramuscular and subcutaneous adipose tissue in Laiwu pig was verified qRT-PCR. *p < 0.05; **p < 0.01; ***p < 0.001.

According to the original detection results of Real Time PCR and the relative quantitative calculation formula of 2^−△△CT^, we calculated the relative quantitative results of the indirect target genes of each sample, as shown in Fig. 8. The gene standard curve and dissolution curve showed that R2 reached 0.99, indicating that the linear correlation was very high, and the amplification efficiency was above 90%, indicating that the experiment was effective (Supplementary material). The q-PCR results are consistent with the sequencing results. This indicates that the sequencing results are true and reliable.

## Discussions

In this experiment, L_PX was used as the control group, and L_JN was the experimental group. A total of 265 differentially expressed circRNAs were identified, of which 178 were up-regulated and 87 were down-regulated. Since most circRNAs come from exon regions within protein coding genes, circRNA can inhibit the expression of host genes^39^. Since the circular structures of circRNAs covalently linked are stable, they have a longer half-life. Most circRNAs perform biological functions by acting as molecular sponges for miRNAs^44^. Therefore, we annotated the host gene function of DEcircRNA, the molecular sponge of miRNA, and ceRNA regulation networks, and used the circRNA-miRNA-mRNA axis to analyze the potential regulatory functions of circRNA.

In this study, we annotated the host genes of circRNA, and found that the host genes were mainly involved in SMAD protein complex and other GO terms related to fat deposition and metabolism. The result of KEGG enrichment analysis showed that host genes were mainly involved in TGF-beta signaling pathway.

The phosphorylation of the SMADs family can activate TGF-β Signaling pathway^45^. TGF-β can inhibit the differentiation of 3T3-F442A cells into adipocytes through SMAD3 signal pathway^46^. The transforming growth factor β (TGF β) superfamily consists of more than 33 members, including t TGF βs, BMPs, GDFs, activating, and nodule-associated proteins^47^. BMPs and TGF-β bind to the receptor protein to form complexes that specifically activates and phosphorylates SMADs protein. The phosphorylated SMADs protein binds with SMAD4 to form complexes and transfer to the nucleus, thereby regulating gene transcription^46^. TGF-β-SMAD2/3 signal can stimulate the proliferation of preadipocytes and inhibits fat adipogenesis. It can mediate bile acid and phospholipid metabolism after hepatotropic acid liver injury^48^. The BMP-SMAD1/5/8 signal pathway can not only promote the proliferation of adipocytes, but also promote adipogenesis^46^. The host genes of circRNA was involved in above signaling pathways to regulate adipogenic differentiation and fat metabolism.

In addition to the ability of circRNA to regulate the transcription of host genes, more studies have shown that circRNA performs its regulatory function by competitively binding miRNAs. In this study, we predicted the binding relationship between DEcircRNA and miRNA, and found that circRNA_06424 and circRNA_08840 can bind more miRNA, as shown in Fig. 4.

CircRNA_06424 can competitively bind with 25 known miRNAs and inhibit the function of miRNAs. The expression level of miR-149 was significantly increased in a mouse model of non-alcoholic fatty liver disease induced by a high-fat diet^49^. Further studying the functions of miR-149, we found that miR-149 can competitively bind *FGF-21* to promote fat production in HepG2 cells^50^. MiR-149 is up-regulated in IMF, and may play a role in promoting fat deposition in IMF cells. It can be used as a candidate marker molecule for high intramuscular fat pig breeds without affecting the level of subcutaneous fat deposition.

A total of 43 miRNAs were predicted to bind to circRNA_08840, miR-331-3p, ssc-miR-128, miR-361-3p, miR-143-5p, miR-874 and other miRNAs are differentially expressed in L_PX and L_JN. Chen et al. used Laiwu pig adipocytes as experimental materials to explore the function of miR-331-3p, and found that miR-331-3p can inhibit the proliferation of adipose precursor cells by affecting the expression of key cell cycle genes, such as *CD2*, *CD3*, and *CD4*. In addition, it promotes the differentiation of pre-adipocytes and promotes fatty acid synthesis by regulating *DLST* to regulate the citric acid-pyruvate cycle^51^. CircRNA_08840 can competitively bind miR-331-3p to regulate the proliferation and differentiation of precursor fat and fatty acid deposition. The expression level of miR-128 in the plasma of type 2 diabetic patients was significantly increased^52^. Pan et al. transfected porcine preadipocytes with over-expressing miR-128 plasmid under glucocorticoid treatment (with miR-SC and miR-128 inhibitors as the control group). The results showed that the level of triglycerides was significantly increased. The further analysis showed found that miR-128 caused lipid accumulation by inhibiting the expression of *SIRT1*^53^. The expression of miR-128 was down-regulated in L_JN. This is consistent with the results of existing studies. Wang et al. selected Huainan pig and Duroc as experimental materials and constructed a ceRNA regulatory network. They found that miR-874 is closely related to adipogenesis^54^. circRNA_08840 can regulate the level of lipid deposition through binding with target miRNAs.

The results showed that some miRNAs, such as miR-328, miR-296-5p, miR-365-5p, miR-339-5p, and miR-774, can simultaneously bind to circRNA_08840 and circRNA_06424. It has been found that metabolic disorders can present in cancer patients, especially in lipid metabolism. Therefore, some scholars believe that abnormally metabolized lipids are markers of cancer^55^. Numerous studies have shown that miR-328 exerts s the anti-tumor effect by regulating target genes and downstream pathways^56,57^. The overexpression of miR-328-3p can aggravate the oxidative stress injury of endothelial cells in the arterial valve^58^. MiR-328 can reduce inflammation, apoptosis, and oxidative stress of endothelial cells induced by oxidized low-density lipoprotein (Ox-LDL) by specifically binding to *HMGB1*^59^. Overexpression of miR-328 can inhibit the expression of *PTEN* and inhibit the activation of PI3K-Akt signaling pathway^60^. The activity of PI3K-Akt signal pathway was decreased, and glucose absorption was decreased, thereby regulating lipid metabolism. The overexpression of miR-328 can inhibit *BACE1* translation, increase glucose metabolism, promote lipid accumulation in muscle progenitor cells, and promote the differentiation of brown adipocytes and brown adipocytes^61^. In human adipose hepatocytes, miR-296-5p can target the 3'UTR of *PUMA* and reduce the expression of *PUMA*, thereby reducing lipotoxicity and adipocyte apoptosis^62^. Belarbi compared the activity of *EBF1* and differentially expressed miRNA in the white fat (WAT) of obese women and non-obese women. They found that mi-365-5p was expressed at a high level in obese women’s WAT and could target and regulate *BEF1* and overexpress miR-365-5p can reduce the activity of *EBF1* and cause white fat hypertrophy^63^.

In this study, we performed functional annotation analysis of the ceRNA network centered on circRNA_06424 and circRNA_08840. The results showed that the indirect target genes of circRNA_06424 were mainly involved in the positive regulation of phosphatidylinositol 3-kinase signaling, phosphatase inhibitor activity, fatty acid binding, and other related GO terms related to lipogenesis and metabolism. The results of KEGG showed that the indirect target genes of circRNA_06424 were mainly involved in signaling pathways, such as Biosynthesis of unsaturated fatty acids, PPAR signaling pathway, and Wnt signaling pathway. These signaling pathways are classic signaling pathways that regulate fat deposition metabolism. This is similar to the results of the functional annotation analysis of the ceRNA regulatory network with circRNA_08840. Wnt signal pathway was divided into classical Wnt signal pathway and atypical Wnt signal pathway^64^. Typical Wnt signaling pathway can enhance preadipocyte proliferation^65^ and inhibit the differentiation of preadipocytes into adipocytes^66^. The atypical Wnt signaling pathway can inhibit PPAR transcription in bone marrow mesenchymal stem cells, thereby inhibiting the differentiation of mesenchymal stem cells into adipocytes^67^. However, some studies have shown that atypical Wnt signal pathway ligand *WNT5B* can promote adipogenesis by inhibiting the typical Wnt signaling pathway when adipogenic precursors are stimulated by adipogenesis^68^.

We have verified the function of ceRNA network, which can play an important regulatory role in lipogenesis and metabolism. Similarly, we found that the indirect target gene of novel circle RNA is at a key position in the PPI network, especially *PPARD*..Osmotic proliferator-activated receptor family (PPARs) are lipid-activating factors that play multiple roles in fat development and metabolism. The main function of *PPARD* is to directly or activate related genes to regulate adipogenesis and lipid metabolism. *PPARD* agonists in mice can alleviate obesity caused by high-fat diet. In vitro experiments, activating *PPARD* in adipocytes can promote fatty acid oxidation and utilization^69^. Overexpression of *PPARD* in brown adipose tissue increases lipid droplet consumption.^69^. It can also induce the expression of *UCP1* (a marker gene of brown fat). *PPARD* can also participate in fatty acid metabolism in skeletal muscle^70,71^. Mouse myoblasts overexpressing porcine *PPARD* can inhibit the formation of myotubes in vitro. When *PPARD* and *PPARG* are co-expressed, they can promote the expression of fatty acid-binding protein (aP2) and other adipogenic genes in the cell, resulting in a large accumulation of triglycerides^72^. Cytokines secreted by adipocytes can induce *PPARD* expression, thereby inducing alternate activation of macrophages^73^. Adipocytes and macrophages use intercellular mitochondrial transfer to participate in the metabolic homeostasis, including promoting the glucose utilization of by adipocytes, regulating lipid storage, and increasing energy consumption^74^. *PPARD* directly enhances the transcriptional activity of *MMP9* and promotes umbilical vein endothelial cell (EPCS) and C2C12-grade cell differentiation by activating the *PPARD*-*MMP9*-*IGF-1* axis^75^. However, EPCS and C2C12 were cultured in vitro under certain specific conditions with the potential to differentiate into intramuscular fat^76,77^. *MMP9* can degrade the basement membrane^78^, thereby promoting the invasion of macrophages into fat cells. In addition, the expression of *MMP9* was correlated with the level of low-density lipoprotein sterol^79^, lipid metabolism and lipid uptake^80^, visceral obesity^81^, atherosclerotic lesions^82^, diabetes and tumors^83^, and other lipogenesis and metabolism diseases. The research on *PPARD* shows that the single nucleotide polymorphism (SNP) of *PPARD* has a great impact on its function^84,85^. For example, RS6902123 is an SNP significantly associated with type 2 diabetes^86^. In this study, compared with other genes, the expression of *PPARD* was higher. And the expression of *PPARD* in SCF was significantly higher than that in IMF. The amount of IMF and SCF in Laiwu pigs were significantly higher than that of other breeds of pigs. *PPARD* has different mechanisms of action on fat deposition in different parts of Laiwu pigs, which may be related to the interaction of related genes and SNP^87^. Nonetheless, the exact mechanism of action is uncertain. *PPARD* plays an important role in fat deposition^87^. This is consistent with the results of existing studies. CircRNA_06424 can regulate the expression of *PPARD* through the competitive binding of miRNA with the mRNA. This is the reason for the different regulatory mechanisms of *PPARD* in IMF and SCF.

## Conclusions

In sum, this study used circRNA sequencing and bioinformatics technology to analyze the expression of circRNAs in the subcutaneous and intramuscular adipose tissues of Laiwu pigs for the first time. A total of 265 differentially expressed circRNAs were identified in the subcutaneous adipose tissue. Among them, 178 were up-regulated and 87 were down-regulated in the subcutaneous adipose tissue. CircRNA–miRNA target prediction was performed, and a circRNA–miRNA–mRNA interaction network was constructed. Through GO and KEGG pathway analyses, we found that these indirect target genes and host genes of differentially expressed circRNAs were involved in adipogenic differentiation and lipid metabolism, as well as disease-related pathways. This indicates that circRNAs can regulate adipogenic differentiation and lipid metabolism. This study provides a theoretical basis for further study on the mechanism of fat deposition, providing potential therapeutic targets for metabolism related diseases.

## Supplementary Information

Supplementary Figures.

## Data Availability

All data were presented in the main manuscript and are available to readers.

## Abbreviations

RNA-seq: RNA sequencing technology
GO: Gene ontology
KEGG: The Kyoto Encyclopedia of Genes and Genomes
IMF: Intramuscular fat
SCF: Subcutaneous fat
DEcircRNAs: Differentially expressed circRNAs
DEmiRNAs: Differentially expressed miRNAs
DEgenes: Differentially expressed target genes of miRNA
PCR: Polymerase chain reaction
PPI: Protein–protein interaction
