# Supplementary material for: The comprehensive detection of miRNA and circRNA in the regulation of intramuscular and subcutaneous adipose tissue of Laiwu pig
Source: Sci Rep. 2022 Oct 3;12:16542. doi: 10.1038/s41598-022-21045-2 (PMC9530237; doi:10.1038/s41598-022-21045-2)
Supplement: Supplementary file 1 — Supplementary Figures. [file 41598_2022_21045_MOESM1_ESM.docx]

**Figure 1 circRNA_00907 gene standard curve and product dissolution curve**

| **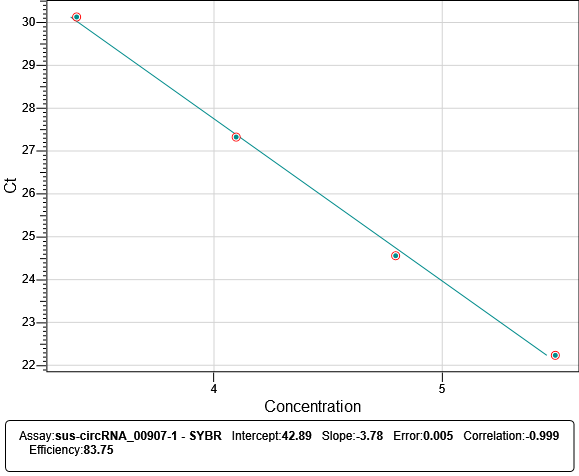** | **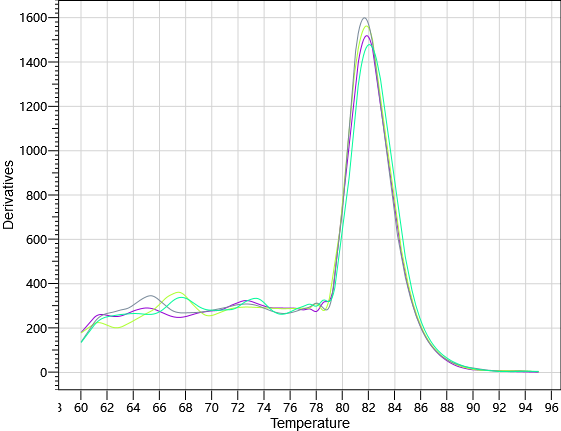** |
| --- | --- |

**Figure 2 circRNA_01106 gene standard curve and product dissolution curve**

| **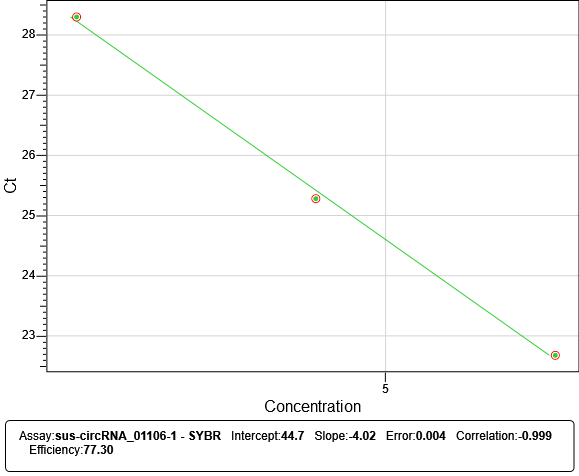** | **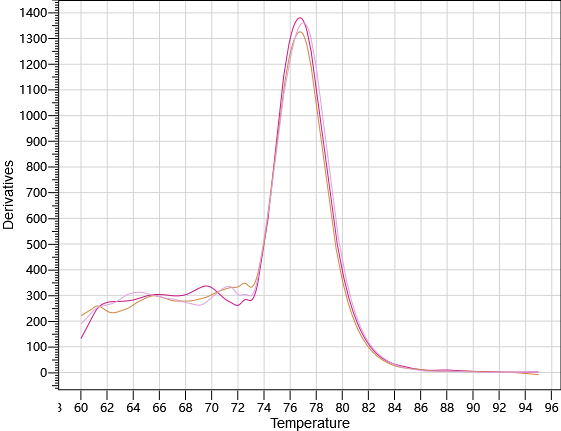** |
| --- | --- |

**Figure 3 circRNA_06424 gene standard curve and product dissolution curve**

| **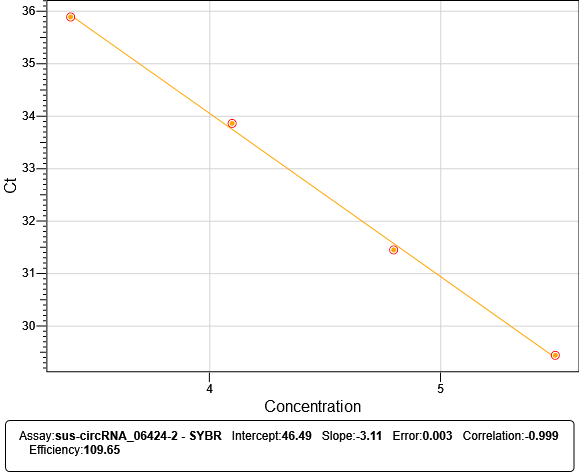** | **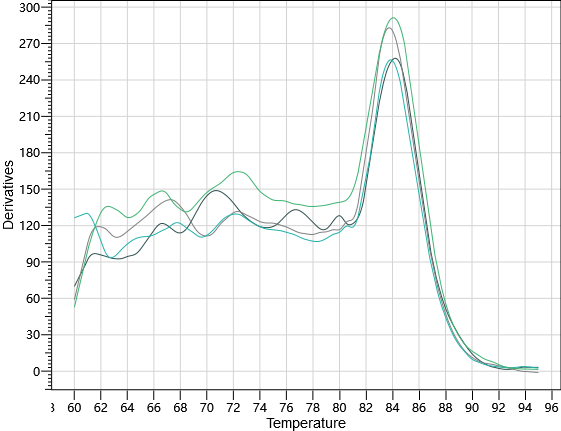** |
| --- | --- |

**Figure 4 CircRNA_08840 gene standard curve and product dissolution curve**

| **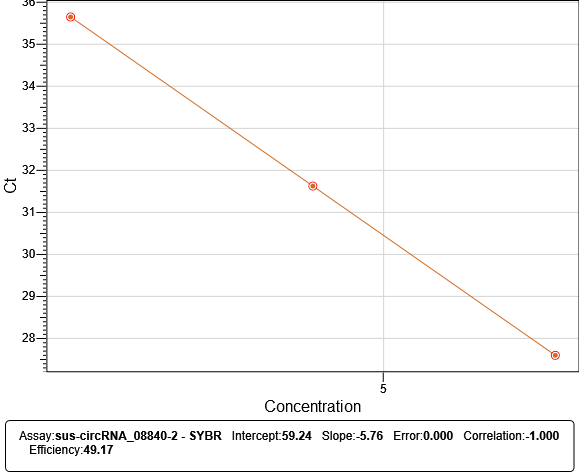** | **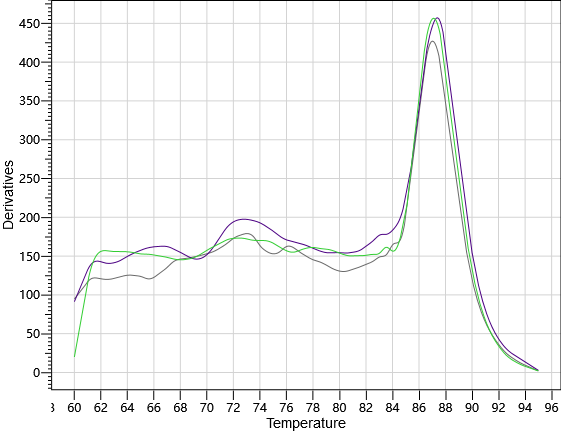** |
| --- | --- |

**Figure 5 CircRNA_15332 gene standard curve and product dissolution curve**

| **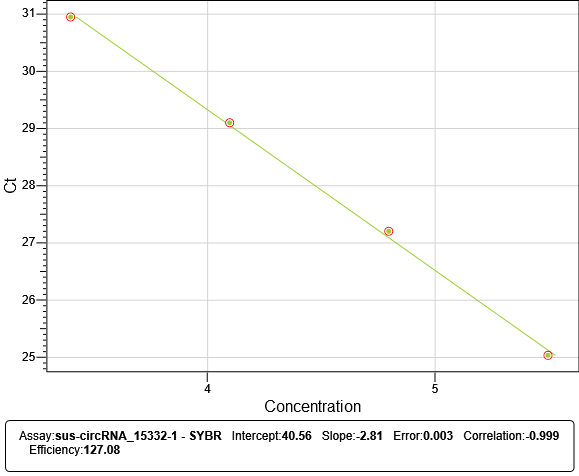** | **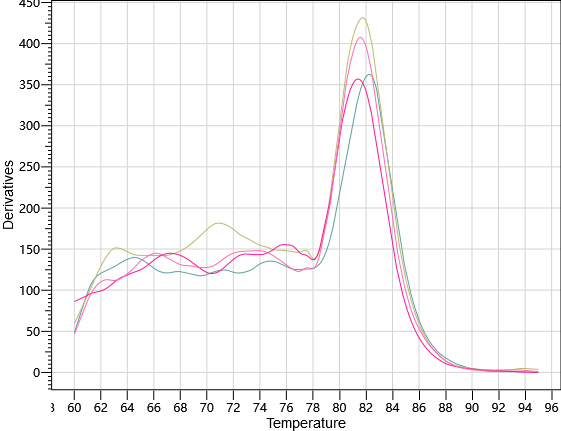** |
| --- | --- |

**Figure 6 CircRNA_22410 gene standard curve and product dissolution curve**

| **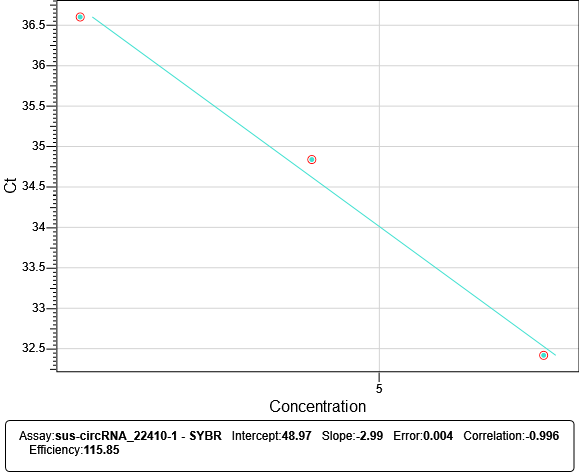** | **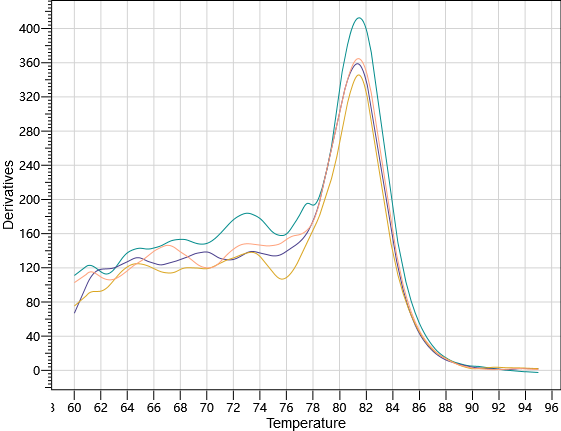** |
| --- | --- |

**Figure 7 miR-361-3p gene standard curve and product dissolution curve**

| **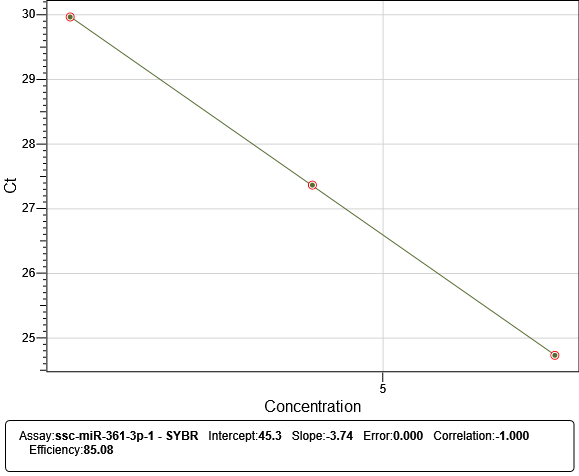** | **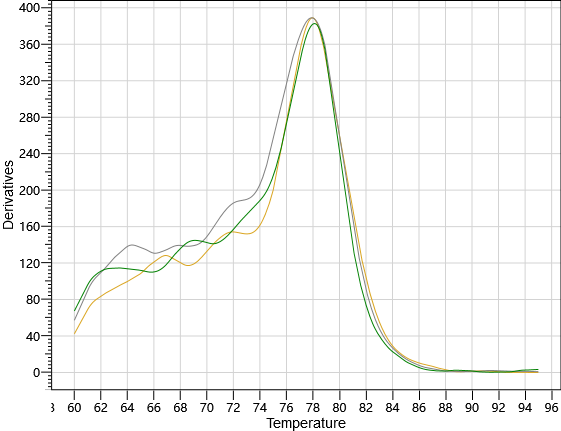** |
| --- | --- |

**Figure 8 miR-331-3p gene standard curve and product dissolution curve**

| **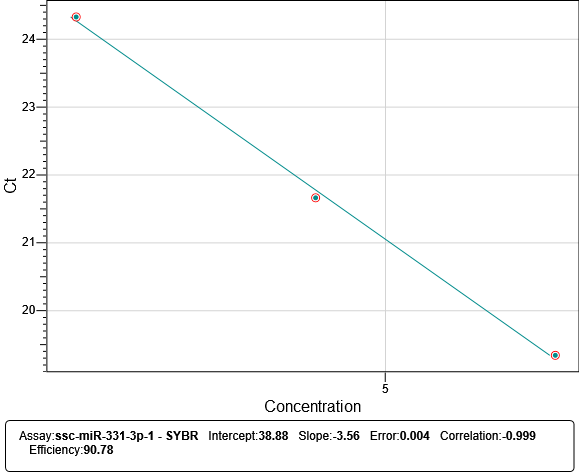** | **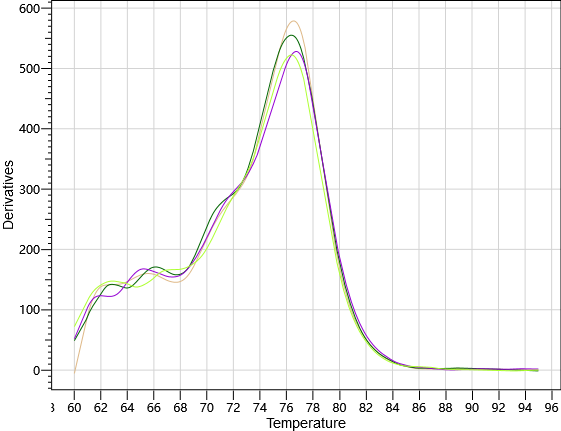** |
| --- | --- |

**Figure 9 miR-328 gene standard curve and product dissolution curve**

| **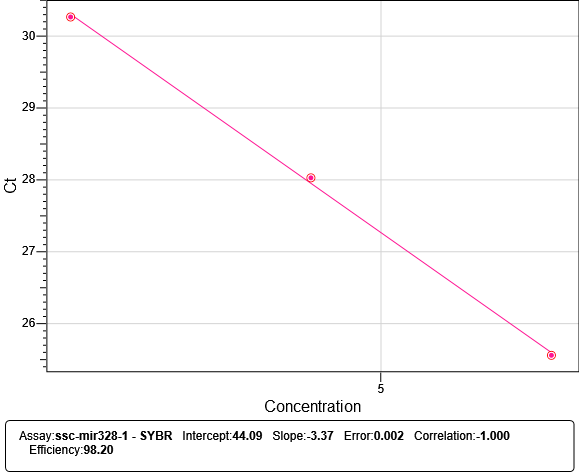** | **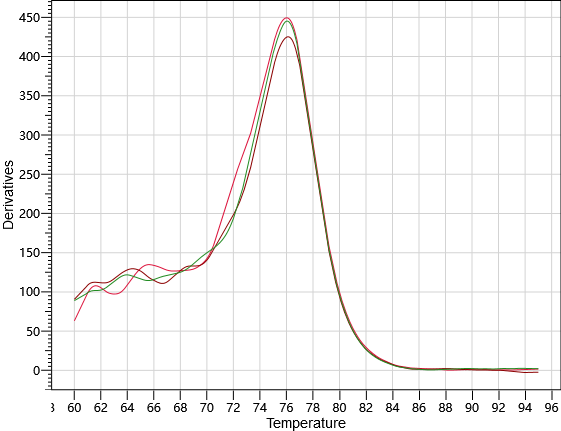** |
| --- | --- |

**Figure 10 PPARD gene standard curve and product dissolution curve**

| **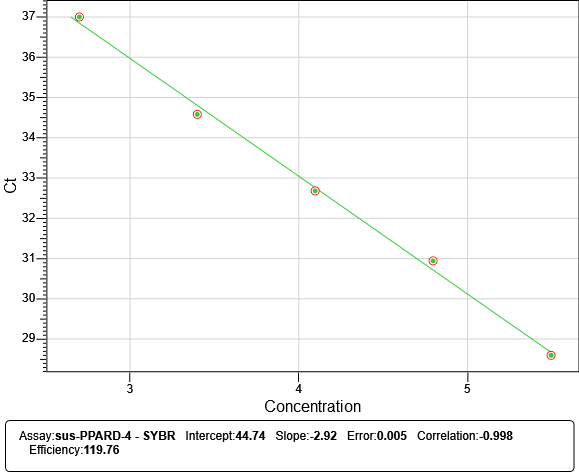** | **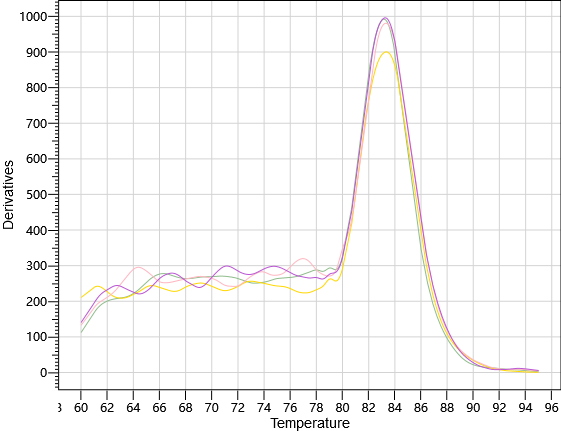** |
| --- | --- |

**Figure 11 MMP9 gene standard curve and product dissolution curve**

| **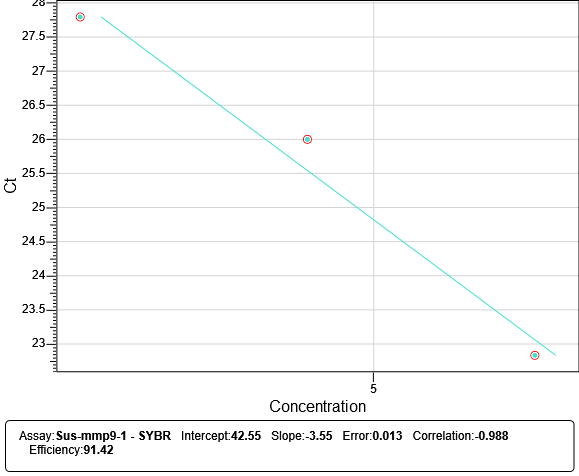** | **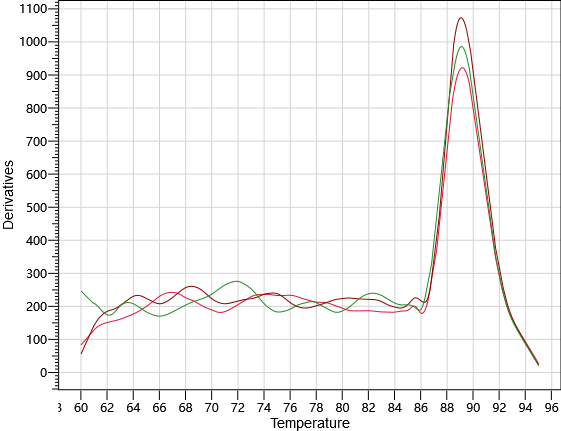** |
| --- | --- |
